# CRISPR-Cas-Mediated Gene Silencing Reveals RacR To Be a Negative Regulator of YdaS and YdaT Toxins in *Escherichia coli* K-12

**DOI:** 10.1128/mSphere.00483-17

**Published:** 2017-11-22

**Authors:** Gargi Bindal, Revathy Krishnamurthi, Aswin Sai Narain Seshasayee, Devashish Rath

**Affiliations:** a Molecular Biology Division, Bhabha Atomic Research Centre, Mumbai, India; b Homi Bhabha National Institute, Training School Complex, Anushakti Nagar, Mumbai, India; c National Centre for Biological Sciences, Tata Institute of Fundamental Research, Bangalore, India; d Shanmuga Arts, Science, Technology & Research Academy, Thanjavur, Tamil Nadu, India; University of Iowa

**Keywords:** CRISPR gene silencing, *Escherichia coli*, RacR, essential gene, *rac* prophage, toxin

## Abstract

Bacterial genomes are rich in horizontally acquired prophages. *racR* is an essential gene located in the *rac* prophage that is resident in many *Escherichia coli* genomes. Employing a clustered regularly interspaced short palindromic repeat (CRISPR)-Cas-based gene silencing approach, we show that RacR is a negative regulator of the divergently transcribed and adjacent *ydaS-ydaT* operon in *Escherichia coli* K-12. Overexpression of YdaS and YdaT due to RacR depletion leads to cell division defects and decrease in survival. We further show that both YdaS and YdaT can act independently as toxins and that RacR serves to counteract the toxicity by tightly downregulating the expression of these toxins.

**IMPORTANCE** *racR* is an essential gene and one of the many poorly studied genes found on the *rac* prophage element that is present in many *Escherichia coli* genomes. Employing a CRISPR-based approach, we have silenced *racR* expression to various levels and elucidated its physiological consequences. We show that the downregulation of *racR* leads to upregulation of the adjacent *ydaS-ydaT* operon. Both YdaS and YdaT act as toxins by perturbing the cell division resulting in enhanced cell killing. This work establishes a physiological role for RacR, which is to keep the toxic effects of YdaS and YdaT in check and promote cell survival. We, thus, provide a rationale for the essentiality of *racR* in *Escherichia coli* K-12 strains.

## Abstract (precis)

*racR* is an essential gene and one of the many poorly studied genes found on the *rac* prophage element that is present in many *Escherichia coli* genomes. Employing a CRISPR-based approach, we have silenced *racR* expression to various levels and elucidated its physiological consequences. We show that the downregulation of *racR* leads to upregulation of the adjacent *ydaS-ydaT* operon. Both YdaS and YdaT act as toxins by perturbing the cell division resulting in enhanced cell killing. This work establishes a physiological role for RacR, which is to keep the toxic effects of YdaS and YdaT in check and promote cell survival. We, thus, provide a rationale for the essentiality of *racR* in *Escherichia coli* K-12 strains.

## INTRODUCTION

Bacterial strains within a species often show high genetic and phenotypic diversity. A survey of the *Escherichia coli* genome reveals conservation of a small percentage of genes, whereas most other genomic regions are variable (1). Horizontal gene transfer mechanisms have contributed significantly to the evolution of bacterial genome plasticity. Transposable elements, plasmids, and prophages are a major source of intra- and interspecies genetic diversity. It is estimated that *E. coli* K-12 has gained 1,600 kb of new genetic material, a significant portion being nine cryptic prophages which comprise 3.6% of its genome, since its divergence from *Salmonella* sp. (2). The perpetuation of horizontally acquired genetic elements depends upon whether the selective advantage conferred outweighs the fitness cost of its maintenance. Novel genes contained within chromosomally integrated prophages can introduce beneficial phenotypes that confer a selective advantage to the hosts. It can allow the bacteria to thrive in a competitive environment and successfully occupy the niche. This is exemplified by prophages that express adaptive bacterial immune systems known as clustered regularly interspaced short palindromic repeat (CRISPR)-Cas systems (3, 4). Some of the well-studied genes that are of phage origin and are especially beneficial to pathogens are determinants for virulence, resistance, and tolerance to antibiotics.

RacR, a putative repressor, is encoded by the defective *rac* prophage which has 24 genes and 5 pseudogenes (5). While products of some genes belonging to this prophage have been shown to be involved in functions like recombination, motility, and controlling biofilm formation (6), most other genes belonging to the *rac* prophage are not functionally well characterized. RacR is predicted to be a DNA binding transcriptional DNA regulator (7, 8), but its *in vivo* function and physiological significance remain unknown. *racR* is included in a set of about 299 genes that could not be deleted and are considered essential in *E. coli* (5, 9).

As a knockout or deletion of *racR* was not feasible, we exploited a CRISPR-based gene silencing approach to manipulate expression of *racR*, an essential gene carried by the *rac* prophage of *E. coli* K-12, to show that RacR depletion has a profound effect on growth and morphology of the cells. We further show that *racR* silencing causes significant growth arrest and cell death. We present evidence to show that RacR is a negative regulator of the adjacent and divergently transcribed *ydaS-ydaT* operon and that increased expression of these toxins causes growth inhibition and morphological defects in cells. Our findings suggest that RacR promotes cell survival by modulating the expression of YdaS and YdaT (YdaS/T) toxins.

## RESULTS

### Cascade-mediated silencing of *racR.*

**FIG 1:**
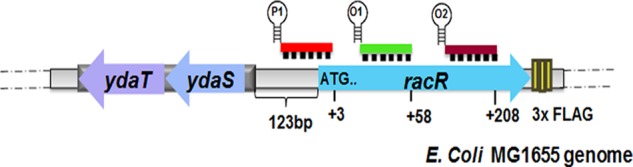
Schematic representation of the *rac* prophage region indicating Cascade binding positions on the *racR* promoter and the ORF (not to scale). P1 is the crRNA targeting the promoter, O1 is the crRNA targeting the ORF proximal to the translation start site, and O2 is the crRNA targeting the ORF distal to translation start site.

**FIG 2:**
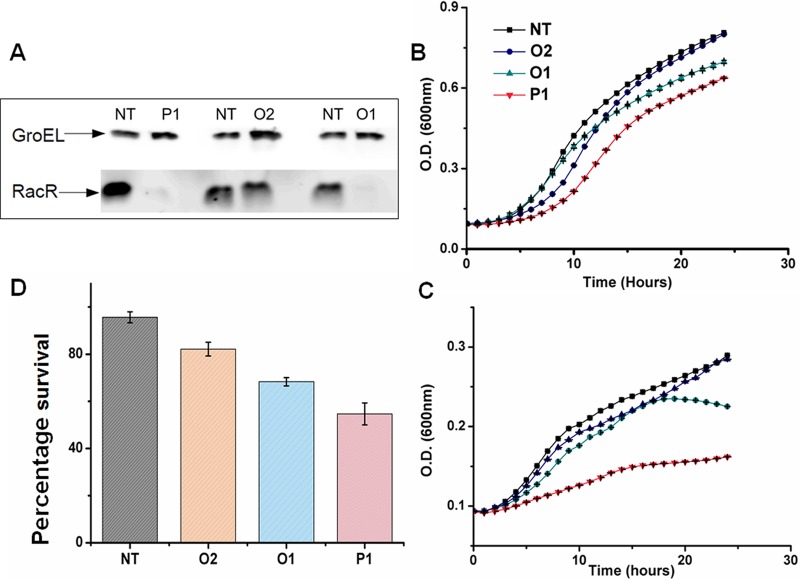
Growth defect caused by RacR depletion. (A) RacR levels in cells 5 h after induction of Cascade-based transcriptional silencing. (B and C) Growth in the absence (B) and presence (C) of inducer. Growth is measured by the optical density at 600 nm. (D) Cell survival after 5 h of induction of the *racR* silencing. NT refers to the nontargeting control crRNA. P1, O2, and O1 refer to crRNAs targeting the different regions of *racR* (Fig. 1).

To implement the Cascade-based silencing system against the *racR* gene in *E. coli*, we modified the type IE system of *E. coli*. In this system, the different Cas proteins combine with a short RNA (CRISPR RNA [crRNA]) to form a surveillance complex (Cascade) that binds to the target DNA based on the sequence complementarity with the crRNA. Upon binding to the target DNA, the Cascade complex recruits Cas3 nuclease that destroys the target DNA (10). In the absence of Cas3 nuclease, the crRNA can be manipulated to direct the Cascade to bind to a DNA target of choice, e.g., a promoter, to interfere with transcription, which leads to silencing (11). The Cascade protein complex from *E. coli* was expressed from a T7 promoter, while the crRNA was placed under the isopropyl-β-d-thiogalactopyranoside (IPTG)-inducible P_LlacO-1_ promoter (11). As *racR* deletion is known to be lethal, we designed crRNAs containing spacers complementary to different regions of the *racR* gene to achieve different levels of silencing (Fig. 1). We expected to achieve higher level of silencing by targeting the promoter, which would interfere with transcription initiation, compared to targeting within the open reading frame (ORF), which is expected to interfere with the transcription elongation step. However, as the promoter of *racR* is not defined, a spacer was designed to target the intergenic region between the divergently transcribed *racR* and *ydaS* genes. Care was taken to position this spacer as close as possible to the translation start site of *racR*. Two additional spacers targeting different positions in the coding sequence of *racR* were designed. To test whether the Cascade-crRNA complex could efficiently repress *racR* expression on the genome, an *E. coli* strain carrying the FLAG-tagged *racR* gene on the genome was constructed. The silencing of *racR* was measured by determining the level of RacR protein using anti-FLAG antibody for each crRNA plasmid. Upon induction of the CRISPR system, compared to the control (nontargeted crRNA plasmid), the crRNA against the putative *racR* promoter region significantly knocked down gene expression, whereas targeting other adjacent regions on the ORF showed only a moderate effect (Fig. 2A). Even in uninduced samples, the promoter-targeting spacer showed reduced RacR levels compared to a nontarget spacer, suggesting that a low level of leaky expression of the CRISPR system was sufficient to give an observable silencing effect (see Fig. S1 in the supplemental material).

10.1128/mSphere.00483-17.1 FIG S1  RacR levels in cells competent for *racR* silencing in the absence of induction of the silencing system. NT refers to the nontargeting control crRNA. P1, O2, and O1 refer to crRNAs targeting different regions of *racR* (Fig. 1). Download FIG S1, PDF file, 0.1 MB. Copyright © 2017 Bindal et al. 2017 Bindal et al. This content is distributed under the terms of the Creative Commons Attribution 4.0 International license.

### Reduced levels of RacR lead to growth defects.

To test whether Cascade-mediated downregulation of RacR results in a growth defect, we performed time course measurements of growth in the presence and absence of the inducer. In the absence of the inducer, cells carrying crRNAs targeting *racR* showed differential but modest growth defect (Fig. 2B). In the presence of the inducer, the growth defect was more pronounced with cells harboring the promoter targeting crRNA showing an extreme growth defect compared to control cells with nontargeting crRNA (Fig. 2C). While targeting the promoter region significantly reduced growth, targeting the transcribed region affected growth moderately. Together, these results suggested that silencing of *racR* has a growth inhibitory effect where the extent of inhibition is proportional to the level of expression of the RacR protein. Further, even a small perturbation in the RacR level, for example, due to leaky expression of the silencing system, is sufficient to cause a growth defect. Survival was checked at different time points after induction of *racR* silencing, and the results (Fig. S2) indicated a significant reduction in survival after 5 h. Hence, subsequent analyses were carried out after 5 h of induction of silencing.

10.1128/mSphere.00483-17.2 FIG S2  Survival at different time points after induction of RacR silencing. NT refers to cells expressing nontargeting crRNA, and P1 refers to cells expressing crRNA targeting the promoter region of *racR* (Fig. 1). The cultures were induced, and aliquots taken at different time points were serially diluted (10^−1^- to 10^−6^-fold). Appropriate dilutions were spotted on LB agar plates, and the plates were incubated overnight at 37°C. Download FIG S2, PDF file, 0.2 MB. Copyright © 2017 Bindal et al. 2017 Bindal et al. This content is distributed under the terms of the Creative Commons Attribution 4.0 International license.

### Cell survival is dependent upon RacR expression.

As silencing of *racR* led to poor growth of cells, the effect of *racR* silencing on cell survival was investigated. Live and dead cell populations were quantified after the induction of silencing machinery for all the crRNA plasmids. The quantification was done by flow cytometry using propidium iodide (PI) as the marker for dead cells and SYBR green I dye as a counterstain. Measurements after 5 h of induction showed that silencing of *racR* expression leads to significant cell death (Fig. 2D). The level of cell survival was again dependent on the extent of silencing of *racR* with higher silencing leading to higher cell death. For example, with crRNA targeting the *racR* promoter, cell survival was only about 50%, while for O2 crRNA which is targeted to the C-terminal half of the ORF, the survival improved to about 80%.

### RacR depletion leads to gross morphological changes.

**FIG 3:**
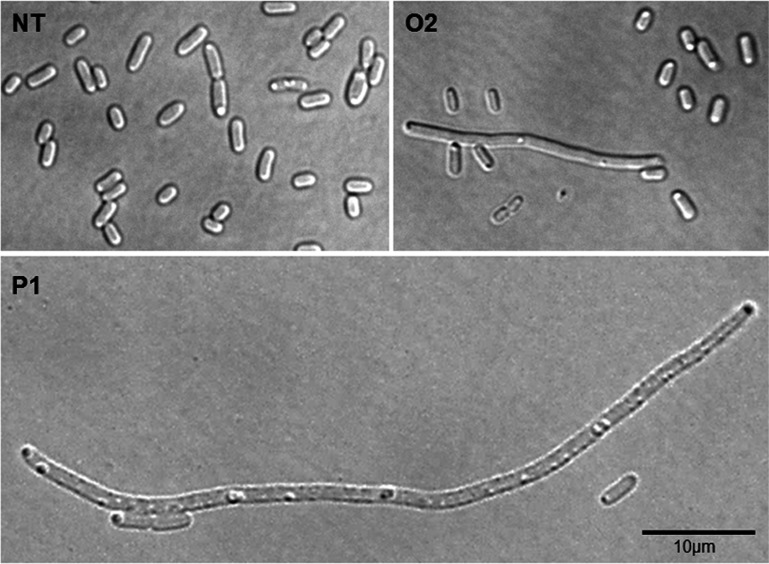
Morphological changes associated with *racR* silencing. Cells observed by bright-field microscopy (magnification of ×100). NT refers to the nontargeting control crRNA. P1 and O2 refer to crRNAs targeting different regions of *racR* (Fig. 1).

To further assess the effect of silencing, the cells carrying different crRNAs targeting *racR* were visualized with a microscope after induction of silencing. While control cells and cells expressing a nontargeting crRNA were indistinguishable and showed normal size and shape, *racR* silencing caused striking morphological changes in cells (Fig. 3). Most cells in which *racR* was silenced exhibited copious filamentation as well as an increase in the cell diameter (Fig. 3). The number of cells exhibiting filamentation and the extent of elongation were greater when P1 crRNA was used than when O2 crRNA was used, indicating increased growth abnormalities with increases in silencing of *racR*. The cells were also examined after staining with 4′,6′-diamidino-2-phenylindole (DAPI) and Nile red which stain the DNA and lipids, respectively. The DNA was observed to be distributed throughout the filament suggesting that while replication of DNA was not affected, cell division was severely hindered (Fig. S3). *racR* silencing-induced filamentation strongly suggests that the cell death associated with *racR* silencing could be due to perturbed cell division.

10.1128/mSphere.00483-17.3 FIG S3  Representative images illustrating morphological changes associated with *racR* silencing after 5 h of induction. Cells were treated with DAPI and Nile red stains to visualize the nuclear material and the membrane, respectively. NT refers to cells expressing nontargeting crRNA, and P1 refers to cells expressing crRNA targeting the promoter region of *racR* (Fig. 1). Download FIG S3, PDF file, 0.1 MB. Copyright © 2017 Bindal et al. 2017 Bindal et al. This content is distributed under the terms of the Creative Commons Attribution 4.0 International license.

### Silencing of *racR* upregulates *ydaS* and *ydaT* expression.

**FIG 4:**
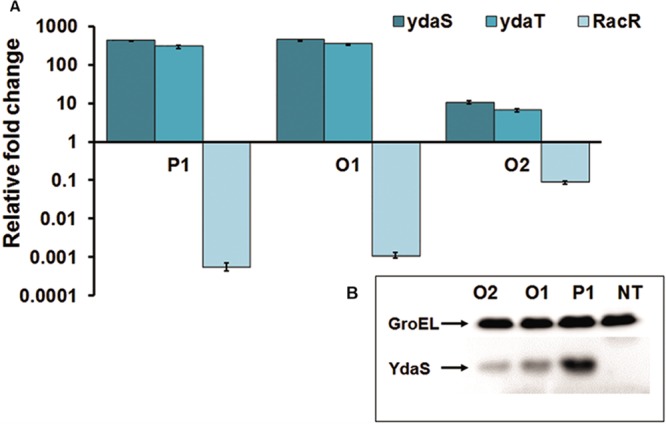
Change in the relative expression profiles of *ydaS* and *ydaT* in response to *racR* downregulation. (A) Fold change in expression of *racR*, *ydaS*, and *ydaT* 5 h after induction of the silencing of *racR* (by targeting P1, O1, and O2 crRNAs) relative to their expression in nontargeting control cells (NT crRNA) as determined by RT-qPCR. (B) Western blot using anti-FLAG antibody showing FLAG-tagged YdaS levels in cells expressing either nontargeting control crRNA (NT) or crRNAs (P1, O2, and O1) targeting the different regions of *racR* (Fig. 1).

*ydaS* and *ydaT* form an operon and are divergently transcribed from the promoter region of *racR*. To gauge the impact of Cascade-based silencing of *racR* on its possible downstream targets, we simultaneously measured transcript levels of *racR* and of *ydaS* and *ydaT* by quantitative real-time PCR (RT-qPCR) (Fig. 4). After 5 h of induction of silencing machinery, *racR* transcript levels decreased significantly, with cells expressing promoter-targeting crRNA showing the highest (approximately 1,000-fold) reduction (Fig. 4A). Similarly, targeting the *racR* ORF led to expected reductions in transcript levels mirroring the RacR protein profile under these conditions (Fig. 2A). Remarkably, *ydaS* and *ydaT* expression showed an inverse correlation with *racR* expression. The fold change in expression of *ydaS* as well as *ydaT* was proportional to the fold repression of RacR expression, indicating that RacR is a negative regulator of YdaS and YdaT (YdaS/T).

To analyze whether upregulation of *ydaS* transcription, in turn, resulted in elevated protein levels, a strain carrying FLAG-tagged *ydaS* which replaced the native *ydaS* gene on its genome was constructed. *racR* silencing was induced in this strain by expressing Cascade along with individual crRNAs, and YdaS levels were determined using anti-FLAG antibody. The results showed a significant increase in YdaS levels in response to RacR depletion (Fig. 4B). As expected, YdaS levels correlated well with the level of depletion of RacR. These results combined with the fact that *ydaS* and *ydaT* have been annotated as a toxin-antitoxin pair in the RASTA server (12) suggested the following two possibilities. (i) RacR is a regulator of the YdaS/T toxin-antitoxin system. (ii) One or both of YdaS/T could be a toxin, and RacR may provide a function similar to an antitoxin.

### Differential contribution of YdaS and YdaT to cell toxicity and morphological defects.

**FIG 5:**
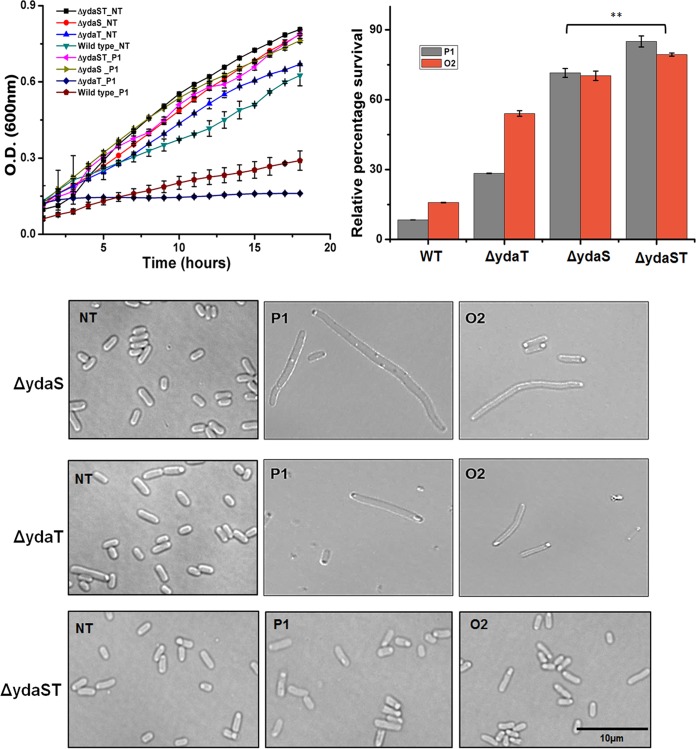
Differential effects of *ydaS* and *ydaT* on the morphology and survival of cells in response to *racR* silencing. (Top left panel) Growth curves of wild-type and mutant strains with *racR* silencing. (Top right panel) Percentage survival of wild-type (WT) and mutant strains relative to the nontarget control 5 h after induction of *racR* silencing. Values that are significantly different (*P* value of <0.02 by *t* test) are indicated by a bracket and two asterisks. Values are means ± standard errors of the means (error bars) from four independent trials. (Bottom panel) Morphological changes in Δ*ydaS*, Δ*ydaT*, and Δ*ydaST* cells after 5 h of induction of *racR* silencing. NT refers to the nontargeting control crRNA. P1, O2, and O1 refer to crRNAs targeting different regions of *racR* (Fig. 1).

In order to assess which one of the two (YdaS or YdaT) acts as a toxin or whether one of these two proteins serves as a cotoxin to the other, *ydaS* and *ydaT* were individually deleted. We also generated a double deletion Δ*ydaS* Δ*ydaT* strain. All three strains were viable but had different growth patterns when *racR* was silenced in these strains. To silence *racR*, plasmids expressing Cascade and individual crRNAs targeting *racR* were transformed into Δ*ydaS*, Δ*ydaT*, and Δ*ydaS* Δ*ydaT* cells, and growth of these strains was monitored under inducing conditions (Fig. 5, top left panel). Both the wild-type strain and the Δ*ydaT* strain expressing P1 crRNA showed severe growth retardation, while the Δ*ydaS* Δ*ydaT* strain carrying the P1 crRNA showed no growth defect. This amelioration of growth defect in the Δ*ydaS* Δ*ydaT* background proves that the toxic effect of *racR* silencing is mediated through *ydaS* and* ydaT*. The fact that both Δ*ydaS* and Δ*ydaT* strains were viable ruled out the possibility that *ydaS* and *ydaT* alone could act as a toxin-antitoxin pair.

*racR* silencing induced cell killing as well as filamentation in both the Δ*ydaS* and Δ*ydaT* strains but not in the Δ*ydaS* Δ*ydaT* strain (Fig. 5, top right and bottom panels). However, the extent of the effect was different for each strain. Silencing of *racR* in a Δ*ydaT* background resulted in a more pronounced decrease in survival, while in Δ*ydaS* cells, the effect on survival was less severe but significant (Fig. 5, top right panel). Interestingly, the deletions had a contrasting effect on cell filamentation. Most of the Δ*ydaT* cells which showed the cell division defect were arrested at the two-cell stage, while Δ*ydaS* cells showed copious filamentation (Fig. 5, bottom panel) somewhat similar to that seen upon *racR* silencing in a wild-type background (Fig. 3). These results show that both YdaS and YdaT contribute to toxicity and morphological defects. The fact that RacR silencing irrespective of the presence or absence of YdaS causes cell filamentation implicates YdaT as the protein that has a major effect on cell division. YdaS, on the other hand, seems to be the protein predominantly contributing to cell killing.

### *racR* silencing does not affect biofilm formation.

As it has been previously reported that deletion or excision of the *rac* prophage increases biofilm formation (13), we assessed the involvement of *racR* in this phenomenon. Under our experimental conditions, *racR* silencing did not show an observable change in biofilm formation (data not shown).

## DISCUSSION

Every bacterium has a set of essential genes that define its core functions. Usually, a failure to obtain an inactivating mutation or deletion is a good indication of the essentiality of a gene and its function. Apart from the genes that define core functions, certain genes such as the antitoxin gene of a toxin-antitoxin pair may appear as essential as long as they are required to counteract the toxic effect of the deleterious (toxin) gene. In a systematic study where an attempt was made to individually delete every gene, 299 genes of *E. coli* K-12 strain BW25112 were listed whose deletions did not result in viable mutants (9). The list included *racR*, indicating that it is an essential gene. For bacterial genes that are not amenable to genetic deletion analysis, recently described CRISPR-Cas-mediated gene silencing is an attractive approach to study their function (11, 14).

We exploited a type IE CRISPR-Cascade-based gene silencing approach to manipulate the levels of RacR and study the effect of RacR depletion on cell physiology. Cascade-mediated gene silencing was demonstrated earlier in *E. coli* with two heterologous reporter genes such as *gfp* and *bfp* and a few native genes belonging to three operons involved in sugar catabolism (11, 15). However, its utility in studying essential genes has not been demonstrated. We decided to implement a Cascade-based silencing system to repress *racR* expression to achieve two distinct goals; first, to demonstrate Cascade-based silencing for an essential endogenous gene in *E. coli*, and second, and more importantly, to use it as an effective tool to study the effect of *racR* silencing on cell physiology by reducing expression to various levels. By directing the Cascade to bind to distinct regions of *racR*, we could achieve different levels of silencing for *racR*. As *ydaS-ydaT* and *racR* are divergently transcribed from a short intergenic region of 123 bp (Fig. 1) and the promoter of the *racR* gene is not defined, we designed a promoter targeting a spacer upstream of but as close as possible to the translation start site of *racR* to minimally impact the expression of the adjacent *ydaS-ydaT*. The RT-qPCR results (Fig. 4A) and the Western blot analysis (Fig. 4B) show that our design did not affect the expression of *ydaS* negatively. While the colony size and colony morphology did not change, silencing of RacR severely impacted the growth rate of cells in liquid cultures. Cell survival was directly correlated to the extent of depletion of RacR, and cell morphology changed significantly with cells appearing elongated and filamented, suggesting that *racR* silencing had a negative effect on cell division.

Interestingly, in a genome-wide prediction of TA pairs, *ydaS* and *ydaT* were annotated as a toxin-antitoxin (TA) pair in the RASTA server (12) where *ydaS* supposedly expresses a toxin and *ydaT* expresses the antitoxin. However, we propose that both YdaS and YdaT act as toxins, that RacR acts as a negative regulator, and that RacR-mediated downregulation of *ydaS* and *ydaT* is critical for cell survival. We present the following pieces of evidence in support of this hypothesis. A strain in which the *rac* prophage has been deleted has been shown to be viable (16), whereas a strain with the *racR* deletion alone is not viable, indicating that RacR could be an antidote to a toxin that is present on the *rac* prophage itself. While RacR is constitutively expressed in wild-type cells (17), our analysis indicates that YdaS levels are almost undetected in the presence of RacR. The increase in *ydaS* and *ydaT* transcript levels as well as an increase in YdaS protein abundance in proportion to RacR depletion indicates that RacR is a negative regulator of *ydaS* and *ydaT* expression. Direct evidence for this comes from *in vitro* studies (18), which show that RacR binds to the upstream intergenic region and downregulates the expression of *ydaS* and *ydaT*. We could individually delete *ydaS* and *ydaT* which clearly indicated that *ydaS* and *ydaT* alone are not a TA pair. While the toxic effects of *racR* silencing could be observed in Δ*ydaT* (where YdaS levels were elevated) and Δ*ydaS* (where YdaT levels were elevated) backgrounds, the complete alleviation of the toxic effects of *racR* silencing in a Δ*ydaT* Δ*ydaS* double deletion background suggests that YdaS and YdaT both act independently as toxins and that cell survival depends on RacR-mediated transcriptional downregulation of these toxins. This is independently supported by the observation of Krishnamurthi et al. (18) that it is possible to obtain *racR* deletion in Δ*ydaT* Δ*ydaS* background and not in a wild-type background. While it is arguable whether RacR fits into the strict definition of an antitoxin, as evidence for its direct interaction either with the toxin transcript or the toxin protein is lacking, the organization of the genes involved and the net effect of RacR expression suggest that RacR-YdaS/T might serve as an atypical TA system.

On the basis of the nature of antitoxin and mechanisms employed to neutralize the toxin activity, TA systems have been classified into six types (type I to VI) (19). In these systems, the antitoxin, which could be protein or RNA, interacts with the toxin transcript or the toxin protein. In the majority of type II systems, the antitoxin or the toxin-antitoxin complex also regulates the TA operon expression (19). Compared to the above-mentioned systems, two unique features of the RacR-YdaS/T system have been reported in this study. First, RacR is a transcriptional regulator of the expression of toxin, while in all the systems described above, the toxin-antitoxin interaction is a posttranscriptional event as far as the toxin is concerned. Unlike transcriptional repression by antitoxins of type II systems which do not prevent expression of cognate toxins, RacR in a wild-type scenario, almost completely blocks the transcription of toxin. The second unique feature is that there are two toxins involved which are cotranscribed from the same operon and both can act independently of the other. Though YdaS and YdaT differ in the magnitude of toxicity and their effect on cell morphology, the overall effect is similar.

Besides RacR-YdaS/T, the *rac* prophage is known to contain the RalR/RalA TA system (20) and the Kil toxin, an inhibitor of the essential cell division gene *ftsZ* (21). The other cryptic prophage-based TA systems reported in *E. coli* K-12 include RelE/RelB (in Qin prophage) (22), YpjF/YfjZ (in CP4-57) (23), RnlA/RnlB (in CP4-57) (24), YkfI/YafW (in CP4-6) (23), and CbtA/YeeU (in CP4-44) (25). Among these systems, RacR-YdaS/T seems to be novel because of the involvement of two independent toxins. Many of these prophage TA systems are involved in biofilm formation or persister cell formation under stress (19). Preliminary studies show that RacR-YdaS/T is not involved in biofilm formation; however, its involvement in persister cell formation or antibiotic resistance cannot be ruled out, and this will be the subject of future investigations. Though our studies clearly indicate cell division to be a target of the YdaS/T toxin, the exact nature of this interaction needs to be elucidated.

Compared to the traditional approach of toxin overexpression, modulating antitoxin levels by controlled gene silencing could be advantageous to investigate TA systems. Small perturbations in toxin-antitoxin ratios are more likely under natural conditions. Toxin overexpression may overwhelm the cellular machinery which may not be physiologically relevant. The application of CRISPR gene silencing as shown in this study provides an alternative way to investigate newer TA systems and may speed up discovery of systems similar to that of RacR-YdaS/T. The possibility of employing inducible CRISPR silencing provides further flexibility in manipulating toxin-antitoxin ratios.

## MATERIALS AND METHODS

### Bacterial strains and growth conditions.

The *E. coli* K-12 Δ*cas3* MG1655 strain was used as the host strain for the experiments. The *cas3* knockout allele was transferred from strain JW2731 (9) to MG1655 using P1 transduction (26) with kanamycin selection. The kan cassette was removed by expressing FLP recombinase from pCP20 as previously described (27) to generate a marker-less Δ*cas3* MG1655 strain (GB049). The arabinose-inducible T7 RNA polymerase expressing (*araB*::T7RNAP-*tetA*) cassette was transferred into strain GB049 from strain MLS367 by P1 transduction to generate the GB050 strain. To assess RacR and YdaS expression, strain GB050 containing FLAG-tagged *racR* or FLAG-tagged *ydaS* was constructed using the pSUB11 plasmid (28). Δ*ydaS*, Δ*ydaT*, and Δ*ydaS* Δ*ydaT* strains were constructed by a one-step inactivation procedure using the pKD46 plasmid, followed by removal of selection markers using FLP recombinase as described previously (27). The procedure leads to the in-frame deletion of almost the entire open reading frame (ORF) except for six amino acids and the stop codon at the C-terminal end (9). The knockout strains were transduced with T7 RNA polymerase cassette from strain MLS367 by tetracycline selection. Table S1 in the supplemental material lists the *E. coli* K-12 strains used in this work. All strains were routinely propagated in Luria-Bertani (LB) medium and grown with aeration at 37°C. When necessary, the medium was supplemented with kanamycin (30 µg/ml), carbenicillin (100 µg/ml), streptomycin (100 µg/ml), and tetracycline (25 µg/ml). Chromosomal resistance markers were not selected during growth in liquid cultures. Isopropyl-β-d-thiogalactopyranoside (IPTG) (0.1 mM) and 0.2% l-arabinose were added to the medium for induction of expression as required. All of the medium components were purchased from BD Difco, and antibiotics were procured from Sigma-Aldrich.

10.1128/mSphere.00483-17.4 TABLE S1  Bacterial strains used in this work. Download TABLE S1, PDF file, 0.1 MB. Copyright © 2017 Bindal et al. 2017 Bindal et al. This content is distributed under the terms of the Creative Commons Attribution 4.0 International license.

### Plasmid construction and spacer cloning.

For CRISPR RNA (crRNA) expression, repeat sequences flanking different *racR*-specific spacer sequences (P1, O1, and O2 [Fig. 1]) were assembled by designing oligonucleotides and annealing them. The repeat-spacer-repeat cassettes were cloned under the IPTG-inducible P_LlacO-1_ promoter using EcoRI and XbaI sites in pZe12luc (luc stands for luciferase) to obtain different pCRISPR plasmids. A scrambled spacer sequence was used as a nontargeting (NT) control. For expression of Cascade from a T7 promoter, plasmid pWUR400 was used (10). All cloned constructs were verified by sequencing. The silencing machinery was reconstituted in different strains by transforming a specific pCRISPR plasmid along with pWUR400. Oligonucleotides were chemically synthesized by Integrated DNA Technologies (IDT). All enzymes were purchased from New England BioLabs (NEB). See Table S2 and Table S3 for a full list of the plasmids and oligonucleotides, respectively, used in this work.

10.1128/mSphere.00483-17.5 TABLE S2  Plasmids used in this work. Download TABLE S2, PDF file, 0.1 MB. Copyright © 2017 Bindal et al. 2017 Bindal et al. This content is distributed under the terms of the Creative Commons Attribution 4.0 International license.

10.1128/mSphere.00483-17.6 TABLE S3  Oligonucleotides used in this work. Download TABLE S3, PDF file, 0.1 MB. Copyright © 2017 Bindal et al. 2017 Bindal et al. This content is distributed under the terms of the Creative Commons Attribution 4.0 International license.

10.1128/mSphere.00483-17.7 TABLE S4  Sequences of *racR* and CRISPR arrays containing spacers and their associated protospacer adjacent motif (PAM). Download TABLE S4, PDF file, 0.2 MB. Copyright © 2017 Bindal et al. 2017 Bindal et al. This content is distributed under the terms of the Creative Commons Attribution 4.0 International license.

### Western blot.

The cells harboring silencing plasmids, as indicated, were induced for 5 h. The cells were collected by centrifugation and washed three times with 1× phosphate-buffered saline. The cell pellet was lysed in lysis buffer (100 mM Tris [pH 8.0], 20% sucrose, 50 mM NaCl, 10 mM EDTA [pH 8.0], and 1 mg/ml lysozyme), and the total amount of protein was estimated by Bradford reagent (Sigma-Aldrich) per the manufacturer’s instructions. Twenty micrograms of total protein was separated by 15% SDS-PAGE and transferred to a polyvinylidene difluoride membrane. The membrane was probed with a 1:4,000 dilution of anti-FLAG antibody (Sigma-Aldrich) or 1:10,000 dilution of anti-GroEL antibody (Sigma-Aldrich), followed by a 1:10,000 dilution of a horseradish peroxidase (HRP)-conjugated anti-rabbit secondary antibody. Blots were developed by chemiluminescence using LumiLight substrate (Sigma-Aldrich) in accordance with the manufacturer’s instructions and imaged with a Syngene G: box.

### Growth assays.

To obtain growth curves, cultures grown overnight were diluted 1:100 in minimal medium (1× M9 salts, 2 mM MgSO_4_, 0.1 mM CaCl_2_, 10 μg/ml thiamine chloride, 0.4% glycerol, and 0.2% Casamino Acids) with or without inducers in 96-well culture plates (Corning). The cultures were grown for 24 h at 37°C in a BioTek Synergy H1 instrument with continuous shaking. The absorbance at 600 nm was measured after every 1-h interval.

For surviving bacterial counts, strains were grown in LB broth with antibiotics until an optical density at 600 nm (OD_600_) of about 0.2 was reached and then induced for 5 h. After induction, cultures were diluted in saline, and different dilutions were plated on LB agar plates. The number of colonies was counted after overnight incubation at 37°C. The percentage survival was calculated relative to the nontarget control for each strain after normalizing to the cell densities before induction. Each strain was tested with at least three biological replicates.

### Live/dead assay.

For assaying the live and dead cells after induction of silencing machinery, SYBR green I and propidium iodide (PI) were used for double staining of nucleic acids to differentiate the total cell population from dead cells. Overnight cultures were diluted 1:100 in minimal medium with inducers. After 5 h of growth, the cells were harvested by centrifugation and washed with 1× phosphate-buffered saline. The cell pellet was stained with 10-µg/ml PI solution and 1× SYBR green I by incubation at room temperature for 30 min in the dark. The stained samples were washed twice with 1× phosphate-buffered saline. The samples were analyzed with a CyFlow Space-Sysmex Partec flow cytometer with an excitation wavelength of 485 nm and emission wavelengths of 535 nm (green emission) and 635 nm (red emission). At least 100,000 events were recorded for each sample. FlowJo software was used to calculate red-green fluorescence ratios for different pCRISPR plasmids.

### Microscopy.

The cells harboring silencing machinery were harvested after 5 h of induction. The cell pellet was washed once with 1× phosphate-buffered saline. The cells were fixed on a 0.8% agarose pad on the glass slide. For fluorescence microscopy, cells were stained with 4′,6′-diamidino-2-phenylindole (DAPI) (nuclear stain) (1 mg/ml) and Nile red (membrane stain) (1mg/ml) for 1 h at room temperature in the dark. These cells were washed twice with 1× phosphate-buffered saline and fixed on a 0.8% agarose bed on the glass slide. Images were captured with an Olympus IX 83 inverted microscope with a magnification of ×100. The images were processed with ImageJ software, and representative images were used.

### Quantitative real-time PCR.

Total RNA from cells induced for 5 h was isolated using RNAsnap RNA isolation method as previously described (29). The total RNA was treated with 1 U/µl DNase (Invitrogen). Treated RNA samples were used to synthesize cDNAs by RevertAid First Strand cDNA synthesis kit using random primers (Thermo Scientific). The quantitative real-time PCR (RT-qPCR) was performed in triplicate with cDNA samples using KAPA SYBR fast qPCR master mix per the manufacturer’s instructions. The nontemplate and no-reverse-transcriptase controls were included in qPCRs. *rpoD* and 16S rRNA genes were used as reference genes (the gene-specific primers used are listed in Table S3). The samples were run on a LightCycler 480 instrument II (Roche Diagnostics) with the following program. Each sample was heated to 95°C for 3 min, followed by 35 cycles, with 1 cycle consisting of denaturing (10 s at 95°C), annealing (20 s at 52°C), and extension (20 s at 72°C). At the end of the run, a melt curve was generated to ensure the absence of nonspecific products. The efficiency for the primers used was calculated and used to quantify relative gene expression based on the ΔΔ*C*_*T*_ method (30).

### Static biofilm assay.

Biofilm formation was assayed essentially using a protocol described earlier (6) with some modifications. Overnight cultures were diluted 1:100 in LB with or without inducer. Two hundred microliters from a culture was transferred to a flat-bottom 96-well plate (Corning). The plate was sealed properly to prevent evaporation of medium and incubated at 37°C for 48 h without shaking. After the medium was removed and the cells were washed twice with saline, surface-attached cells were covered with 200 µl of 0.2% crystal violet for 30 min. Following two subsequent washes with saline, surface-bound crystal violet was extracted by the addition of 200 µl of acetone-ethanol (80:20) and estimated by absorbance measurements at 570 nm with a BioTek Synergy H1 plate reader.
