# Supplementary figures and images for: CRISPR-Cas-Mediated Gene Silencing Reveals RacR To Be a Negative Regulator of YdaS and YdaT Toxins in Escherichia coli K-12
Source: mSphere. 2017 Nov 22;2(6):e00483-17. doi: 10.1128/mSphere.00483-17 (PMC5700377; doi:10.1128/mSphere.00483-17)

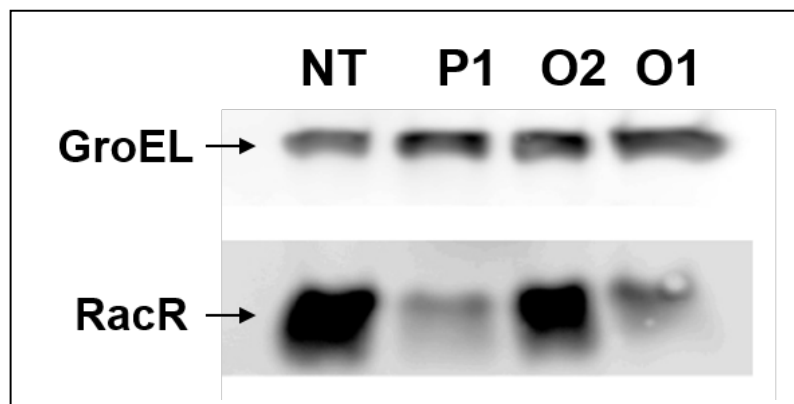

**Fig. S1**

Supplement: FIG S1 [file sph006172408sf1.pdf]

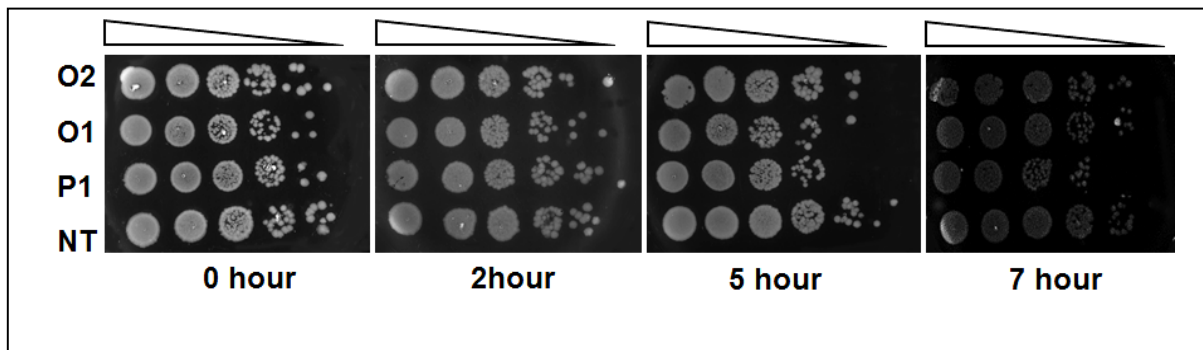

**Fig. S2**

Supplement: FIG S2 [file sph006172408sf2.pdf]

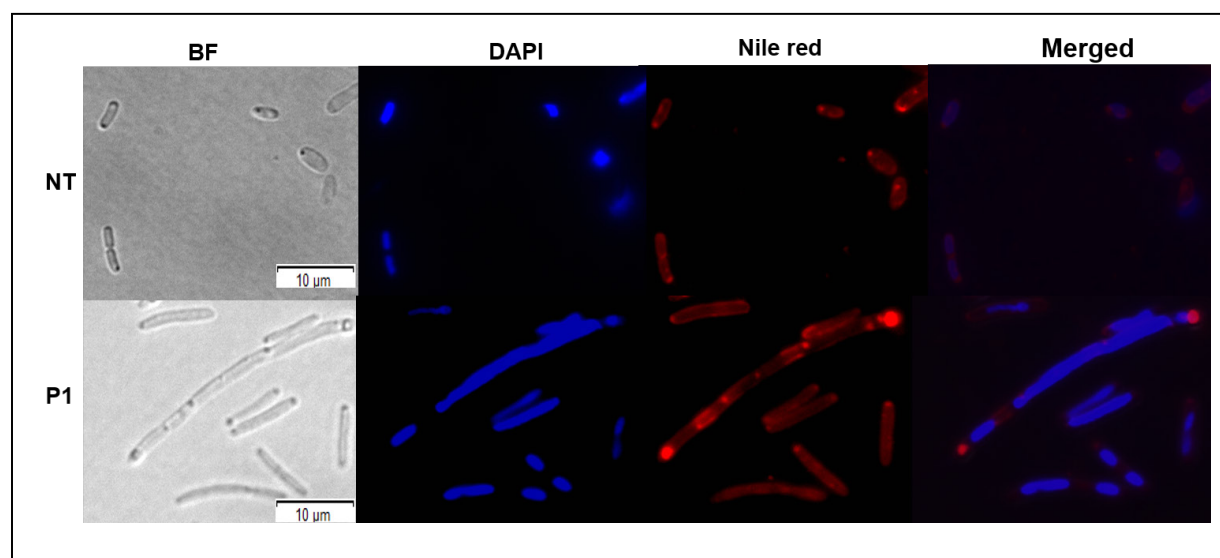

**Fig. S3**

Supplement: FIG S3 [file sph006172408sf3.pdf]
