# Supplementary material for: CRISPR-Cas-Mediated Gene Silencing Reveals RacR To Be a Negative Regulator of YdaS and YdaT Toxins in Escherichia coli K-12
Source: mSphere. 2017 Nov 22;2(6):e00483-17. doi: 10.1128/mSphere.00483-17 (PMC5700377; doi:10.1128/mSphere.00483-17)
Supplement: TABLE S1 [file sph006172408st4.pdf]

Table S1

Strain Name	Genotype	Source
MG1655	*Escherichia coli* K-12 F– λ– *ilvG*– *rfb*-50 *rph*-1	Lab collection
JW2731	BW25113 Δ*cas3::kan*	(1)
GB049	MG1655 Δ*cas3*	This study
MLS367	BW25113 *araB*::T7RNAP-tetA	(2)
GB050	GB049 *araB::* T7RNAP-tetA	This study
GB051	GB050 *racR*:: 3XFLAG	This study
GB052	GB050 *ydaS* :: 3XFLAG	This study
MG1655 Δ*ydaS*	MG1655 Δ*ydaS*	This study
MG1655 Δ*ydaT*	MG1655 Δ*ydaT*	This study
MG1655 Δ*ydaS* Δ*ydaT*	MG1655 Δ*ydaS* Δ*ydaT*	This study
GB053	GB050 Δ*ydaS*	This study

GB054	GB050 Δ*ydaT*	This study
GB055	GB050 Δ*ydaS* Δ*ydaT*	This study

1. Baba T, Ara T, Hasegawa M, Takai Y, Okumura Y, Baba M, Datsenko KA, Tomita M, Wanner BL, Mori H. 2006. Construction of *Escherichia coli* K-12 in-frame, single-gene knockout mutants: the Keio collection. Mol Syst Biol 2:2006.0008.
2. Rath D, Amlinger L, Hoekzema M, Devulapally PR, Lundgren M. 2015. Efficient programmable gene silencing by Cascade. Nucleic Acids Res 43:237–246.
