# Supplementary material for: CRISPR-Cas-Mediated Gene Silencing Reveals RacR To Be a Negative Regulator of YdaS and YdaT Toxins in Escherichia coli K-12
Source: mSphere. 2017 Nov 22;2(6):e00483-17. doi: 10.1128/mSphere.00483-17 (PMC5700377; doi:10.1128/mSphere.00483-17)
Supplement: TABLE S2 [file sph006172408st5.pdf]

Table S2

Plasmid name	Description	Resistance marker	Source
pWUR400	For expression of Cascade from T7 promoter	Streptomycin	(1)
pZE12luc	IPTG inducible $P_{LlacO-1}$ promoter upstream of MCS. For cloning crRNA expressing cassettes	Carbenicillin	(2)
pZE12luc-P1	pZE12luc expressing crRNA with P1 spacer	Carbenicillin	This study
pZE12luc-O1	pZE12luc expressing crRNA with O1 spacer	Carbenicillin	This study
pZE12luc-O2	pZE12luc expressing crRNA with O2 spacer	Carbenicillin	This study
pZE12luc-NT	pZE12luc expressing crRNA with NT spacer	Carbenicillin	This study
pSUB11	For construction of FLAG tagged strain	Kanamycin	(3)
pCP20	Plasmid expressing FLP recombinase	Carbenicillin	(4)

Note: pWUR400 and pZE12luc were a kind gift from Magnus Lundgren, Uppsala University.

1. Brouns SJJ, Jore MM, Lundgren M, Westra ER, Slijkhuis RJH, Snijders APL, Dickman MJ, Makarova KS, Koonin EV, van der Oost J. 2008. Small CRISPR RNAs guide antiviral defense in prokaryotes. Science 321:960–964.

2. Rath D, Amlinger L, Hoekzema M, Devulapally PR, Lundgren M. 2015. Efficient programmable gene silencing by Cascade. Nucleic Acids Res 43:237–246.

3. Uzzau S, Figueroa-Bossi N, Rubino S, Bossi L. 2001. Epitope tagging of chromosomal genes in *Salmonella*. Proc Natl Acad Sci U S A 98:15264–15269.

4. Datsenko KA, Wanner BL. 2000. One-step inactivation of chromosomal genes in *Escherichia coli* K-12 using PCR products. Proc Natl Acad Sci U S A 97:6640-6645.
