# Supplementary material for: CRISPR-Cas-Mediated Gene Silencing Reveals RacR To Be a Negative Regulator of YdaS and YdaT Toxins in Escherichia coli K-12
Source: mSphere. 2017 Nov 22;2(6):e00483-17. doi: 10.1128/mSphere.00483-17 (PMC5700377; doi:10.1128/mSphere.00483-17)
Supplement: TABLE S3 [file sph006172408st6.pdf]

Table S3

Primer Name	Sequence	Description
Tag RacR 3X F	AAGCTCTTGAATCTGAACGGAAAAGCAGAACATCACAAGACTACAAAGACCATGACGG	Construction of 3X FLAG tagged strain
Tag RacR 3X R	GGGGGGGTTAAATAACGGAATCCAGGAGTTTTCCGTCACATATGAATATCCTCCTTAG	Construction of 3X FLAG tagged strain
Tag YdaS 3X F	TGTCAGTGAAGCAACTAAATGACAGTAACAAATCCTCATTTGATCATACCGACTACAAAGACCATGACGG	Construction of 3X FLAG tagged strain
Tag ydaS 3X R	ATTCGATGTGCTCATGCTTGATTTTCATGAATCATTTGCCTCTTGATGTTCATATGAATATCCTCCTTAG	Construction of 3X FLAG tagged strain
ydaS_pkd13_F	GCGTCGCCTAATATTTCTGTGTGTTTTTGGAGTTCATTCGGTGTAGGCTGGAGCTGCTTCG	Construction of knockout strains
ydaS_pkd13_R	CTCATGCTTGATTTTCATGAATCATTTGCCTCTTGATGTTATTCCGGGGATCCGTCGACC	Construction of knockout strains
ydaT_pkd13_ F	CATTTGATCATACCTGAAACATCAAGAGGCAAATGATTCAGTGTAGGCTGGAGCTGCTTCG	Construction of knockout strains

ydaT _pkd13_R	GTGGCTTAGAATAAGCACAAACAGCATGGAAACTTTTGCATTCCGGGGATCCGTCGACC	Construction of knockout strains
racR:3XFLAG forward	TTGCCTAATGTAATGCGCATAGGAGAATATTAAGC	Sequence confirmation of 3X FLAG strain
racR:3XFLAG Reverse	ACGGAATCCAGGAGTTTTCCGTCAGACCATATAAGT	Sequence confirmation of 3X FLAG strain
pZE12luc seq Forward	TAACAATTGACATTGTGAGCGGA	Sequence confirmation of spacer cloning
pZE12luc seq Reverse	GGCCTTTTGCTGGCCTTTTG	Sequence confirmation of spacer cloning
MG1655_16S Forward	AGCCTGATGCAGCCATGCCG	Quantitative RT PCR
MG1655_16S Reverse	AGCCGGTGCTTCTTCTGCGG	Quantitative RT PCR
RT *racR* Forward	TCAAAGGCGGAGGTCGCACG	Quantitative RT PCR
RT *racR* Reverse	GCCCCCAATGCTCTGGACCAA	Quantitative RT PCR
RT *ydaS* Forward	TGTGCTGTTGTCGGTGGGCA	Quantitative RT PCR
RT *ydaS* Reverse	GCTGGACATCTCTCGGCAGGC	Quantitative RT PCR
RT *ydaT* Forward	CTTCGATACCCTGGAACGCC	Quantitative RT PCR
RT *ydaT* Reverse	AGAATCGTCGGAAGAACCGC	Quantitative RT PCR
RT *cspC* Forward	GAATTTTTCATATGGCAAAGATTAAAGGTC	Quantitative RT PCR
RT *cspC* Reverse	ATCAGTGGATCCTATCAGATAGCTGTTACG	Quantitative RT PCR
RT *rpoD* Forward	GACCATCGAGCAGGTTAAAG	Quantitative RT PCR
RT *rpoD* Reverse	CGATGTTGCCTTCCTGAAT	Quantitative RT PCR
