# Supplementary material for: CRISPR-Cas-Mediated Gene Silencing Reveals RacR To Be a Negative Regulator of YdaS and YdaT Toxins in Escherichia coli K-12
Source: mSphere. 2017 Nov 22;2(6):e00483-17. doi: 10.1128/mSphere.00483-17 (PMC5700377; doi:10.1128/mSphere.00483-17)
Supplement: TABLE S4 [file sph006172408st7.pdf]

Table S4

Description	Sequence	PAM
Sequence of *racR* and its upstream intergenic region	TAGTTCTCTTTTTTCATCGAATGAACTCCAAAAACACACAGA<br>AATATTAGGCGACGCCTAACGCAATTGTCAATAGGCTGTGCC<br>TAATGCAGTAAGGGTAGGGATTGCCTAATGTAATGCGCATAG<br>GAGAATATTAAGCA**atg**CTTAGTGGTAAAGACTTAGGCCGAG<br>CGATAGAGCAGGCCATTAACAAAAAAATCGCATCGGGATCCG<br>TCAAATCAAAGGCGGAGGTCGCACGCCACTTTAAAGTCCAAC<br>CACCATCAATTTATGACTGGATTAAGAAAGGCTCTATAAGTA<br>AAGATAAACTTCCAGAATTATGGCGTTTCTTTTCTGATGTTG<br>TTGGTCCAGAGCATTGGGGGCTTAACGAATACCCCATACCAA<br>CCCCCACCAATTCAGATACAAAAAGTGAACTTTTAGATATAA<br>ACAACCTTTATCAAGCAGCCTCTGATGAAATAAGAGCGATTG<br>TAGCTTTCCTGTTATCTGGAAATGCTACAGAACCAGATTGGG<br>TTGACCACGATGTTCGCGCCTACATAGCAGCGATGGAAATGA<br>AAGTGGGTAAGTATCTGAAAGCTCTTGAATCTGAACGGAAAA<br>GCCAGAACATCACAAAAACTGGAACT**taa**	--
CRISPR array containing P1 spacer	GAATTCGAGTTCCCCGCGCCAGCGGGGATAAACCG**GTAACGA**<br>**ATTATAAGAGGATACGCGTAATGTA**GAGTTCCCCGCGCCAGC<br>GGGGATAAACCGTCTAGA	CTT
CRISPR array containing O1	GAATTCGAGTTCCCCGCGCCAGCGGGGATAAACCG**GCTAAAA**<br>**AAACAATTACCGGACGAGATAGCGA**GAGTTCCCCGCGCCAGC	CAT

spacer	GGGGATAAACCGTCTAGA	
CRISPR array containing O2 spacer	GAATTCGAGTTCCCCGCGCCAGCGGGGATAAACCG**GAGACCTGGTTGTTGTAGTCTTTTCTTTGCGG**GAGTTCCCCGCGCCAGCGGGGATAAACCGTCTAGA	CAT
CRISPR array containing NT spacer	GAATTCGAGTTCCCCGCGCCAGCGGGGATAAACCG**CCTTCGCATACGCGCGGCGATACGCTCACGCA**GAGTTCCCCGCGCCAGCGGGGATAAACCGTCTAGA	**----**
